# Permeability-controlled migration of induced seismicity to deeper depths near Venus in North Texas

**DOI:** 10.1038/s41598-022-05242-7

**Published:** 2022-01-26

**Authors:** Kyung Won Chang, Hongkyu Yoon

**Affiliations:** 1 Geotechnology and Engineering Department, Sandia National Laboratories, Albuquerque, 87123 USA; 2 Geomechanics Department, Sandia National Laboratories, Albuquerque, 87123 USA

**Keywords:** Geophysics, Hydrogeology

## Abstract

Migration of seismic events to deeper depths along basement faults over time has been observed in the wastewater injection sites, which can be correlated spatially and temporally to the propagation or retardation of pressure fronts and corresponding poroelastic response to given operation history. The seismicity rate model has been suggested as a physical indicator for the potential of earthquake nucleation along faults by quantifying poroelastic response to multiple well operations. Our field-scale model indicates that migrating patterns of 2015–2018 seismicity observed near Venus, TX are likely attributed to spatio-temporal evolution of Coulomb stressing rate constrained by the fault permeability. Even after reducing injection volumes since 2015, pore pressure continues to diffuse and steady transfer of elastic energy to the deep fault zone increases stressing rate consistently that can induce more frequent earthquakes at large distance scales. Sensitivity tests with variation in fault permeability show that (1) slow diffusion along a low-permeability fault limits earthquake nucleation near the injection interval or (2) rapid relaxation of pressure buildup within a high-permeability fault, caused by reducing injection volumes, may mitigate the seismic potential promptly.

## Introduction

In a poroelastic coupling system, injection of fluids causes two major physical behaviors: (1) Diffusion of pore pressure associated with fluid movement through rock pores and (2) Elastic deformation of rock matrix^1,2^ governed by a system of equations for force equilibrium and transient single-phase flow. This fluid-rock interaction has been suggested as one of the critical mechanisms inducing earthquakes associated with subsurface energy activities such as saltwater/wastewater disposal (SWD)^3–6^, enhanced geothermal stimulation (EGS)^7–9^, and hydraulic fracturing (HF)^10,11^.

The poroelastic coupling effect can be quantified by comparison of spatio-temporal perturbations in total Coulomb stress from the initial state prior to well operations, $\Delta \tau (\mathbf{x} ,t)$ ($\equiv \Delta \tau _s+f\Delta \sigma _n+f\Delta p$, where $\tau _s$, $\sigma _n$, *p* are fault shear and normal tractions and pore pressure, respectively). For typical well injection cases, diffusion-dominant mechanism through permeable formation may nucleate earthquake, if $\Delta \tau$ is sufficient to initiate fault slip, within a pressurized region as a function of distance from the operation location, whereas elastic stress transfer may induce far-field seismicity beyond the pressure fronts^12,13^. Once injection terminates, an immediate decrease in the compressive stress normal to the faults leads to increase in shear stress, and a continuous increase in pore pressure causes sudden increases in $\Delta \tau$. This poroelastic response to the operation phases may explain the surge of post shut-in seismic events, widely observed in the field sites (e.g., SWD in Youngstown, Ohio^14^; and EGS in Basel, Switzerland^15^, Soultz-sous-Forêts, France^16^, and Pohang, South Korea^17^).

Field observations of seismic swarms point out that natural or induced earthquakes nucleate along geological discontinuities (i.e., faults or fracture network) (re)activated by tectonic loading/unloading, fluid flows, and/or deformation of solid earth^18–21^. If faults are oriented favorably to slip or critically-stressed^22^, small perturbations in the pore pressure and stress states driven by multiphysics coupling processes (e.g., poroelastic stressing^23^, hydrochemical dissolution^24^) can overcome frictional resistance to fault slip, potentially nucleating earthquakes along faults. The contrast of hydrological and/or mechanical properties between faults and bounding rocks may enhance or limit either direct impact of pore-pressure diffusion or indirect influence of elastic stress transfer on spatio-temporal patterns of induced earthquakes^25,26^. Higher permeability of the fault can facilitate pore-pressure diffusion to a further distance and a greater depth^27^, and more rigid fault can transmit poroelastic stresses, caused by changes in volume or mass loading, to a deeper fault zone^8^, such that intense accumulation of pore pressure and elastic energy, consistent to large $\Delta \tau$, may induce moderate-to-large magnitude earthquakes ($M_{\text{ w }}\ge 3$) along the fault if $\Delta \tau$ exceeds threshold stress for fault slip. However, physical mechanisms of temporal continuity in rates and magnitudes during/after reduction or cessation of subsurface injection still remain unclear.

Migration of seismicity to deeper depth along the basement faults has been observed commonly in the field sites where a large amount of fluids was injected (e.g., SWD in Oklahoma/Kansas^28^ and Texas^29^). Simple diffusive mechanism may support that continuous injection operations through multiple wells can cause pressure-driven propagation of triggering front of induced seismicity to far-field and deeper depth^30^. However, highly clustered and separated evolution of seismic swarms may involve additional mechanisms, such as Coulomb stress transfer between events^31^, preferential flows driven by heterogeneity of fault/formation properties^32^ or hydraulic connectivity between injection unit and underlying basement^33^ or coexistence of faults with mixed polarity (favorability to slip)^5^. The seismicity rate model indicates that frequency of seismic events of certain magnitude at given background states $R(\mathbf{x} ,t)$ will be determined by Coulomb stressing rate $\dot{\Delta \tau }(\mathbf{x} ,t)$ that can be correlated spatially and temporally to the propagation or retardation of pressure fronts and corresponding poroelastic response to given operation history^12,23^.

In this study, our two-dimensional (2-D) generic study indicates the dominant mechanism perturbing stress states, either diffusion or poroelastic stressing, constrained by the formation permeability. Then, we perform field-scale three-dimensional (3-D) hydro-mechanical coupling simulation of 2015–2018 earthquake sequences observed at the SWD site near Venus, TX in northeast Johnson County. Comparison of $\Delta \tau (\mathbf{x} ,t)$, $\dot{\Delta \tau }(\mathbf{x} ,t)$ and $R(\mathbf{x} ,t)$ to spatio-temporal distribution of 2015–2018 Venus earthquakes will reveal dominant mechanisms inducing seismicity as a function of distance (or depth) from injection operations to the seismogenic fault over time. Sensitivity analyses with variation in the fault permeability are conducted to describe how the permeability-controlled rate of pore-pressure diffusion and stress transfer influences the migration patterns of seismic events along the fault. In addition, the maximum seismic moment and magnitude of potential earthquakes induced by well operations are calculated using either total injected fluid volume or the average total displacement along the fault surface, which will emphasize the importance of site-specific characterization of the seismogenic fault zone for the assessment of seismic hazards associated with subsurface energy activities.

## Impacts of permeability on poroelastic mechanism

Permeability is one of the key hydrogeological factors to control the propagation rate of pressure fronts and corresponding elastic energy transfer that will determine spatio-temporal evolution of dominant mechanism inducing seismicity. Our two-dimensional (2-D) generic study, thus, aims to quantify the impacts of permeability on hydro-mechanical coupled behavior by spatio-temporal perturbation in Coulomb stress components. A 2-D homogeneous domain represents the aerial-view of horizontal section crossing the injection point (Figure S1A), which simulates 30-day injection through a point at the center of the model domain and subsequent shut-in, and implements the aquifer properties given in Table 1. The domain boundaries impose constant pressure and roller conditions, but the extensive length (5 km from the center injection point $\gg 1.7$ km of characteristic diffusive length $\sqrt{4D_a\Delta t}$, where $D_a$ is formation diffusivity) enables to minimize the boundary effect on the hydrological and mechanical behaviors of the formation. To obtain changes in shear and normal tractions driven by injection operation, this model assumes that faults pose the same properties to the background medium, and also, are uniformly distributed throughout the domain with orientation of N-S striking and 60$^\circ$NE. Note that poroelastic response to operations will vary depending on the fault orientation^5,34^, operational constraints (e.g., rate/volume/duration of injection/extraction;^12,35,36^) and/or well design (e.g., number and location of wells;^37^) that can influence spatio-temporal perturbations in stress states and fault stability, such that well operations with gradual rate changes and alignment of multiple wells parallel to seismogenic faults may be the safest strategy to minimize the potential of earthquake nucleation.

**Table 1:** Parameters used in the 2-D generic model.

Poroelastic and transport properties		
	Formation (a)	Fluid (w)
$\kappa _i$ [m$^2$]	1$\times$10$^{-14}$	–
$\phi _i$ [-]	0.25	–
$G_i$ [GPa]	7.6	–
$\nu _i$ [-]	0.15	–
$\nu _{u,i}$ [-]	0.26	–
$\alpha _i$ [-]	0.6	–
$\rho _i$ [kg/m$^3$]	2500	1000
$\eta$ [Pa s]	–	0.4$\times$10$^{-3}$

**Figure 1:**
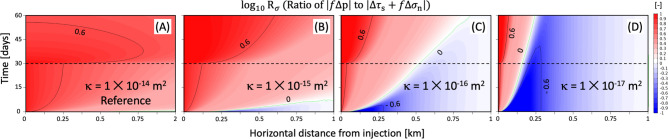
Spatio-temporal distribution of $\text{ log}_{10}R_\sigma$ along the horizontal line of the 2-D model domain (red line in Figure S1A) with variation in the formation permeability ($\kappa =1\times 10^{-14}$ to $1\times 10^{-17}$ m$^2$). The contour of $\text{ log}_{10}R_\sigma =0$ represents $f\Delta p = \Delta \tau _s+f\Delta \sigma _n$, such that diffusion and poroelastic stressing contribute equally to total changes in Coulomb stress.

The relative dominance of pore-pressure diffusion or poroelastic stressing on the change in Coulomb stress is evaluated using the ratio of Coulomb stress components ($R_\sigma$) defined as follows:

1 $$\begin{aligned} \text{ log}_{10}R_\sigma = \text{ log}_{10}\left( \frac{|f\Delta p|}{|\Delta \tau _s+f\Delta \sigma _n|}\right) \left\{ \begin{aligned} >0&\quad \text {diffusion dominant} \\ <0&\quad \text {poroelastic-stressing dominant} \end{aligned} \right. , \end{aligned}$$

where $\text{ log}_{10}R_\sigma =0$ represents that both diffusion and poroelastic stressing contribute equivalently to $\Delta \tau$. Figure 1 shows that spatio-temporal distribution of changes in $\text{ log}_{10}R_\sigma$ with variation in the formation permeability. For the reference case of $\kappa =1\times 10^{-14}$ m$^2$, pore-pressure diffusion increases $\Delta \tau$ dominantly for the whole operational phase. However, immediate poroelastic response to shut-in of injection at $\Delta t=30$ days causes sudden drops in poroelastic stress, which results in rapid increase of positive $\text{ log}_{10}R_\sigma$ indicating stronger influence of delayed diffusion after shut-in (Fig. 1A). For the less permeable formation, confined fluids generate larger $f\Delta p$ and $\Delta \tau _s+f\Delta \sigma _n$ (stronger compression) near the injection point. Low permeability inhibits propagation of the pressure fronts, such that the far-field formation stability will be governed mainly by far-reaching poroelastic effects (negative $\text{ log}_{10}R_\sigma$; Fig. 1B–D). Poroelastic stressing beyond a diffusion-dominant region can slip fault depending on fault orientation or regional faulting stress regime, potentially inducing more seismic events in the distance^5,13^. Slower diffusion expands diffusion-dominant region gradually during the shut-in phase, which may cause post-injection earthquakes as observed in the field sites (e.g., SWD sites in Oklahoma^38^ and Texas, USA^39^, EGS sites in Basel, Switzerland^31^ and Pohang, South Korea^8^).

**Figure 2:**
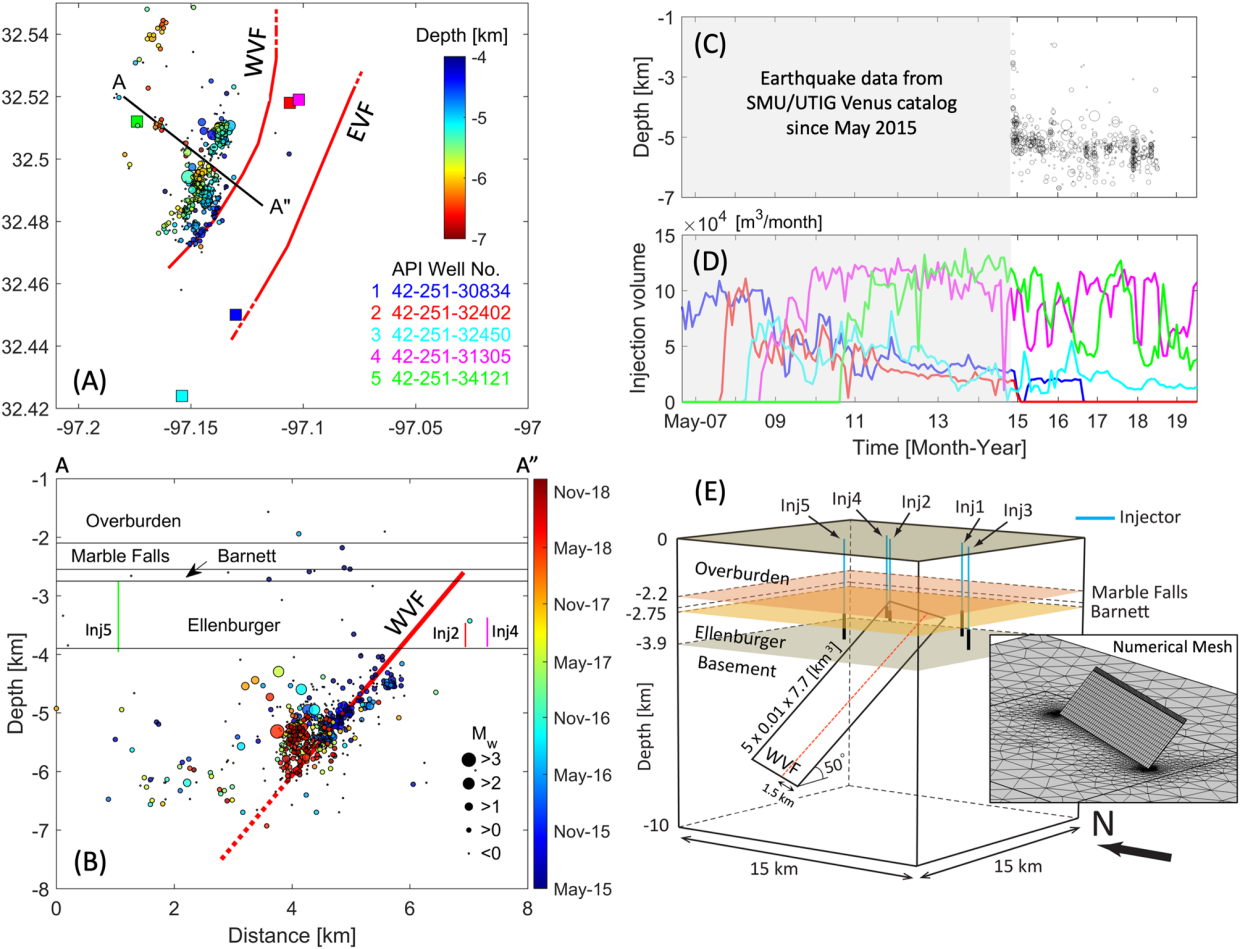
(**A**) Location map of earthquakes and faults near Venus, TX. Faults interpreted on the reflection data are shown at the top of the base- ment (*EVF* Eastern Venus fault; *WVF* Western Venus fault). Five colored squares represent the SWD wells; circles are the earthquake catalog color coded by depth. (**B**) Cross-section along line A–A” with projected WVF, SWD wells and hypocentral locations (circles scaled by magnitude and colored by time). WVF extends to the basement ($\sim$6.1 km of depth) as imaged from the seismic reflection profiles given in^29^. (**C**–**D**) Earthquake catalogs and injection volume through the SWD wells over time. (**E**) Schematic description of 3-D numerical domain including five layers and one fault. SWD wells are modeled as line sources. Cubic meshes are implemented for the fault to solve mechanical behaviors, whereas tetrahedral ones are for the remaining domain. Data of Coulomb stress components are obtained along the orange dash line.

**Figure 3:**
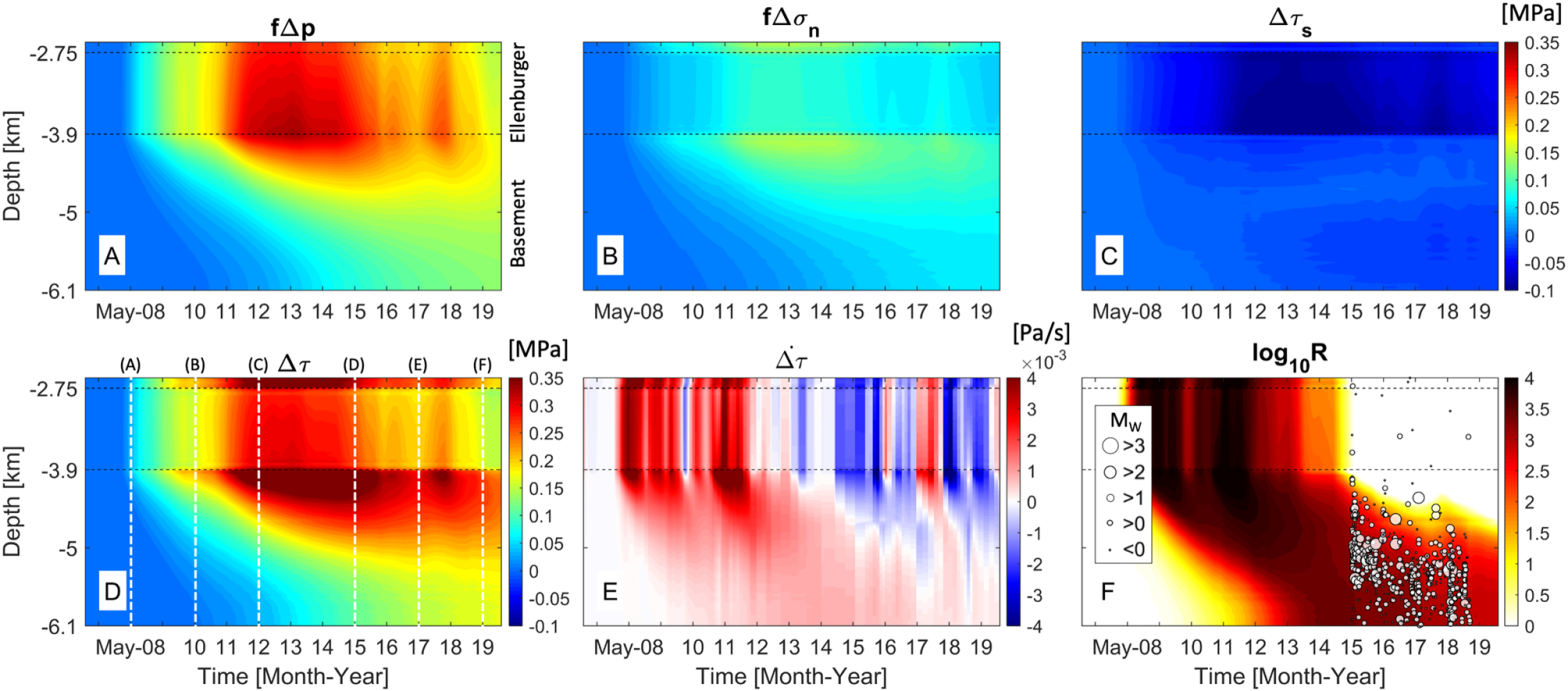
Spatio-temporal distribution of changes in Coulomb stress components (**A**–**C**), total Coulomb stress (**D**), time derivative of Coulomb stress change (**E**), and seismicity rate in base 10 logarithmic scale (**F**) along the fault line indicated as an orange dash line in Fig. 2E. White dash lines in Fig. 3D indicate time steps of the serial results shown in Fig. 4. The earthquake catalogs are fitted to the seismicity rate ($\text{ log}_{10}R$) and the magnitude of earthquakes varies with size of circles.

This generic study indicate that faster fluid flow through a high-permeability formation will expand diffusion-dominant region, favorable to pressure-driven initiation of induced earthquakes, further outward in a shorter period of injection. In a coupled system, poroelastic stressing can impact spatio-temporal perturbations in the pore pressure and stress states beyond the diffusion-dominant region, such that the regime of dominant mechanism will be also function of mechanical characteristics (e.g. rigidity). A more rigid formation requires more elastic strain energy for mechanical behaviors that generates stronger and quicker poroelastic response to injection and subsequent shut-in (Figure S2). In a following section, we extend the generic findings of permeability-controlled poroelastic mechanism to a 3-D field-scale model that simulates pressure and stress perturbations driven by multiple injection operations at the SWD site near Venus, TX in northeast Johnson County, where the pattern of seismic events indicates migrating of induced seismicity to deeper depth over time. The 2-D approach can provide more numerical efficiency with less computational costs, but it restricts diffusion and poroelastic stressing in the 2-D domain which may cause drastic changes of the Coulomb stress components relative to the 3-D model. Therefore, field-scale 3-D modeling with proper configuration of geological and operational characteristics is essential to reveal site-specific physical mechanisms inducing seismicity along finite faults in a layered formation.

## Case study: 2015–2018 Venus Earthquakes

Substantial increases in rates of earthquake occurrence, including local magnitudes of 3.87 and 3.42 in 2015 and 2018 events, have been detected near Venus, TX which may be closely related to SWD operations since 2006^29,40^. This case study aims to reveal the physical mechanisms inducing 2015–2018 Venus earthquakes by implementing operational and geological characteristics of the site into hydro-mechanical coupling simulations, which has not been thoroughly investigated by previous studies. In addition to the Coulomb stress analysis, the seismicity rate model will predict spatio-temporal changes in the potential number of seismic events along the fault using Coulomb stressing rate, $\dot{\Delta \tau }(\mathbf{x} ,t)$, generated from 3-D numerical simulations of multiple injection operations since 2007 (Fig. 2). Injection-induced coupling processes constrained by complexity of fault geometry and depth-dependent heterogeneous/anisotropic features of the fault properties may explain spatio-temporal patterns of observed seismic events^5^, and this study focuses on the role of fault permeability in migration of seismicity into deeper depths.

**Figure 4:**
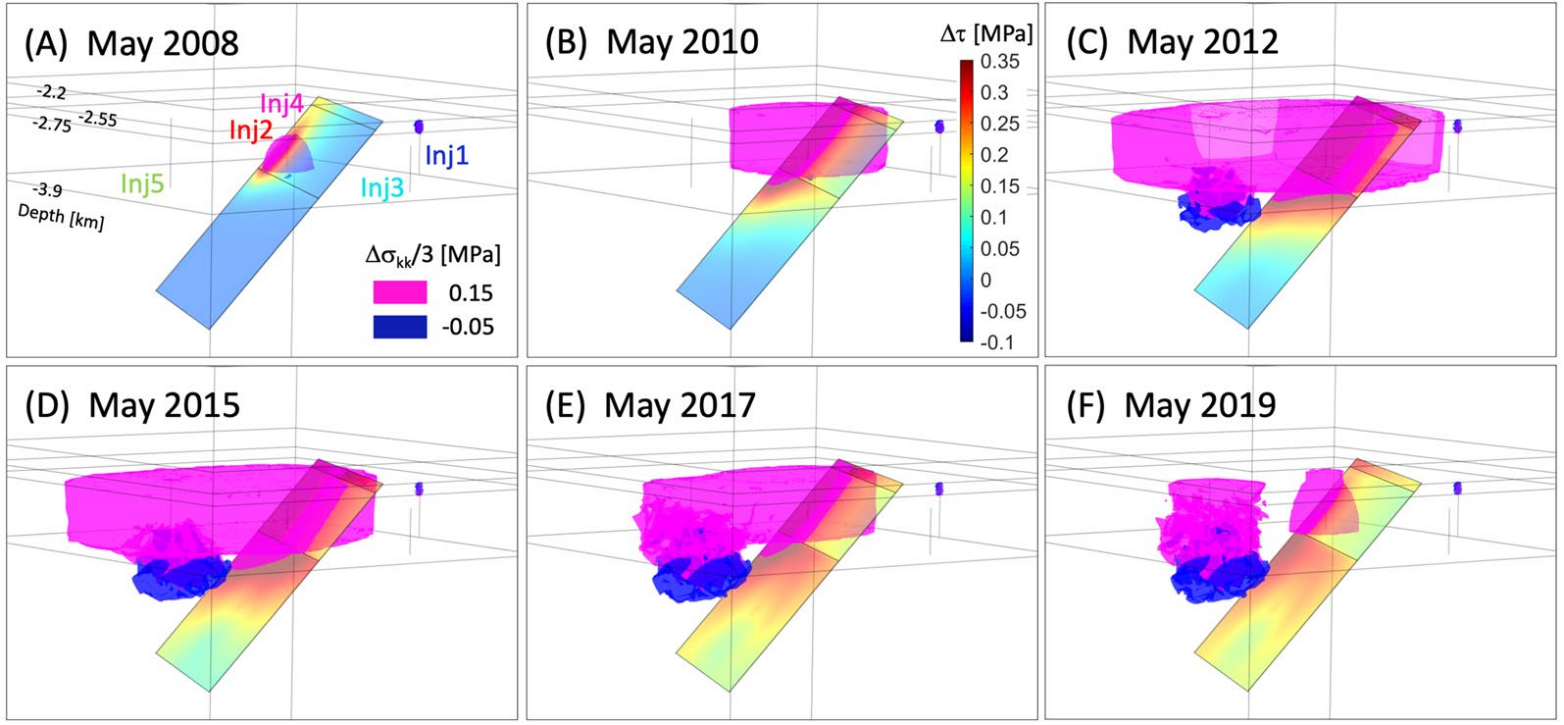
(**A**–**F**) Spatial distribution of isosurfaces of mean stress changes ($\Delta \sigma _{kk}/3$) in the domain and total Coulomb stress change ($\Delta \tau$) along the WVF plane at six time steps. Magenta and blue isosurfaces represent positive and negative mean stress changes of 0.15 and − 0.05 MPa, respectively.

**Figure 5:**
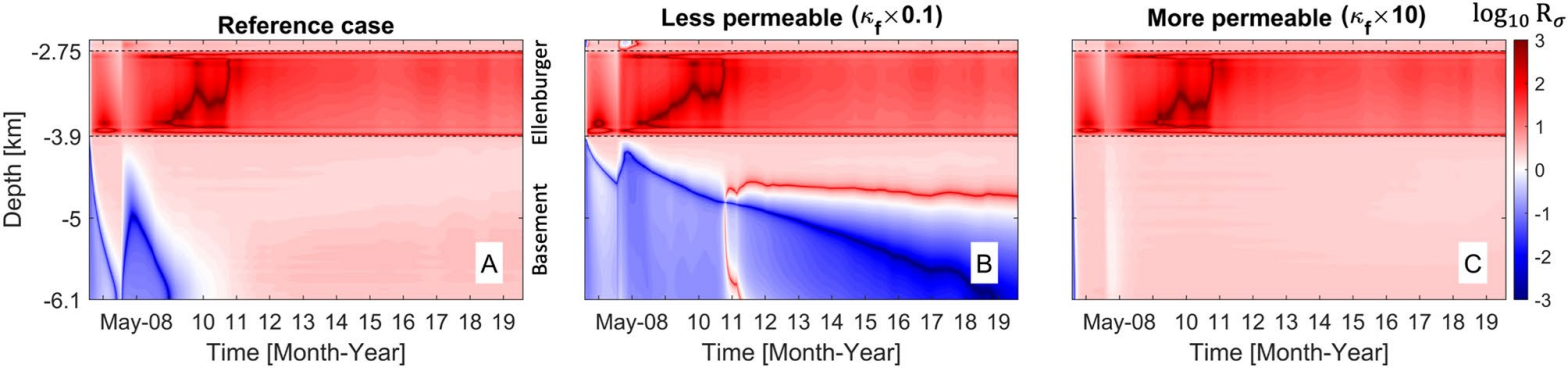
Spatio-temporal distribution of $\text{ log}_{10}R_\sigma$ along the fault line for variation in WVF permeability: (**A**) reference case ($\kappa _f=2.49\times 10^{-15}$ m$^2$) (**B**) less permeable fault ($\kappa _f=2.49\times 10^{-16}$ m$^2$) and (**C**) more permeable fault ($\kappa _f=2.49\times 10^{-14}$ m$^2$).

**Figure 6:**
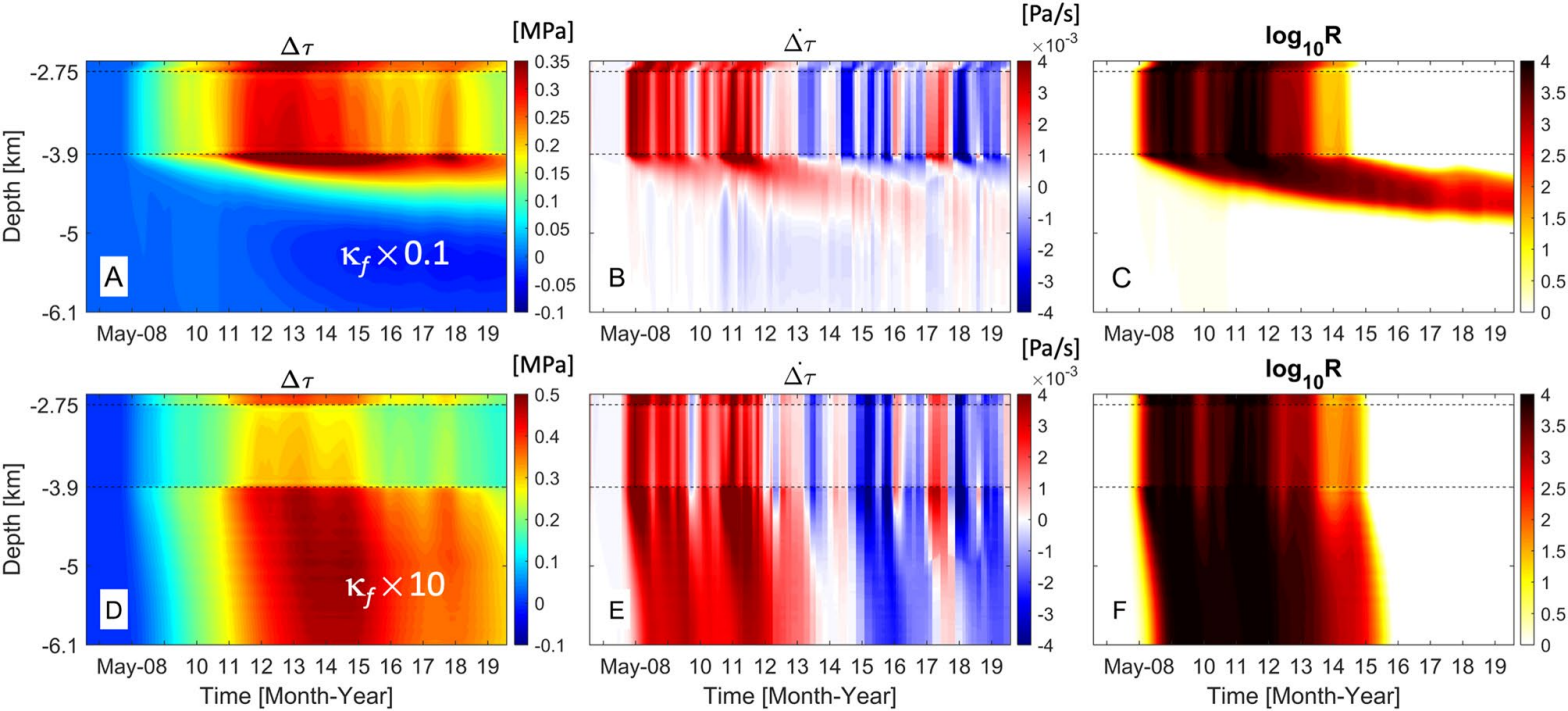
Spatio-temporal distribution of changes in total Coulomb stress, Coulomb stressing rate and seismicity rate in base 10 logarithmic scale along the fault line for variation in WVF permeability: (**A**–**C**) less permeable fault ($\kappa _f=2.49\times 10^{-16}$ m$^2$) and (**D**–**F**) more permeable fault ($\kappa _f=2.49\times 10^{-14}$ m$^2$).

### Causality of spatio-temporal patterns of induced earthquakes

To identify the sequential mechanisms of 2015–2018 earthquake nucleation at the Venus area, we obtain spatio-temporal perturbations in the Coulomb stress components and seismicity rate along the fault line (indicated as an orange dash line in Fig. 2E) from April 2007 to January 2020: changes in pore pressure (Fig. 3A), normal and shear stresses (Fig. 3B,C), total Coulomb stress (Fig. 3D), Coulomb stressing rate (Fig. 3E) and seismicity rate in a base 10 logarithmic scale (Fig. 3F). The *y*-axis represents the upper and lower bounds of the fault zone in a vertical direction, where the fault normal and shear tractions are calculated. The fault line within the southern part of WVF where the majority of seismic events were observed, such that the estimated seismicity rate can be related to physical mechanisms nucleating the seismic events since the observed earthquakes are primarily concentrated near the selected fault line. Different locations of actual seismic events in a lateral direction may indicate the presence of geological complexity (e.g. heterogeneous features or fracture networks along or near the WVF), which is not considered in this study.

The WVF offset stretches from the high-permeability Ellenburger unit to low-permeability basement, and thus, hydraulic characteristics of bounding formations will influence poroelastic response of the fault zone to injection operations^26^. Injection-induced diffusion of pore pressure into/across the fault increase $f\Delta p$ along the WVF bounded by the Ellenburger unit and shallow basement (Fig. 3A). The absolute value of positive $f\Delta p$ varies mainly due to rapid diffusion into/out of the fault zone corresponding to injection operation through Inj4 nearby the fault. Expansion of the injection unit generates positive normal traction that enhances poroelastic stressing on the fault below 3.9 km of depth (Fig. 3B), whereas intense shear stress in the opposite direction of fault slip (negative $\Delta \tau _s$; slip-unfavorable) develops within a diffusion-dominant fault zone between 2.75 and 3.9 km of depth (Fig. 3C), which results in less increase of $\Delta \tau _s+f\Delta \sigma _n$. Distant injection operation through Inj5 pressurizes the injection unit, which will generate poroelastic compression at adjacent basement rocks (Fig. 4C–F; negative mean stress changes in the basement near Inj5), consequently causing slip-unfavorable shear traction (negative $\Delta \tau _s$) along the deep WVF.

Combined effects of direct diffusion and indirect poroelastic stressing lead to larger positive $\Delta \tau$ (up to 0.35 MPa) along the fault zone bounded by basement rocks, not by the Ellenburger unit, as observed between 3.9 and 5 km of depth since 2010 when pressure plumes approach the southern part of WVF (Fig. 3D). Continuous high-volume injection through Inj4 and Inj5 between 2011 and 2015 elevates $\Delta \tau$ significantly along the fault adjacent to the basement. Afterwards, fluctuating injection volumes reduces $\Delta \tau$ in the Ellenburger unit, whereas prolonged diffusion of pore pressure and accumulation of poroelastic stress increase $\Delta \tau$ along the deeper WVF over time. Gradual accumulation of elastic energy along the deep fault zone generate positive Coulomb stressing rate (Fig. 3E), consistent to increases in *R* along deeper WVF over time (Fig. 3F). Hence, more seismic events can occur along the deeper fault zone below 5 km of depth from 2016 to 2018 as spatio-temporal distribution of seismic events matches to the *R*-field.

Figure 4A–F shows the isosurfaces of changes in mean stress ($\Delta \sigma _{kk}/3=\left( \Sigma ^3_{i=1}\Delta \sigma _{ii}\right) /3$) in the model domain and static Coulomb stress changes along the WVF plane at six time steps (indicated as white dash lines in Fig. 3D). Positive and negative $\Delta \sigma _{kk}/3$ represent extensive and compressive stresses, respectively. Prior to injection operation through Inj5, Inj2 and Inj4 operations enlarge the pressurized region, which causes extension (positive $\Delta \sigma _{kk}/3$) within the Ellenburger unit as well as positive $\Delta \tau$ along the permeable fault (Fig. 4A,B). Once injection through Inj5 begins, lateral propagation/retardation of pore pressure generates positive $\Delta \sigma _{kk}/3$ within the high-permeability Ellenburger formation adjacent to WVF, which perturbs stress states along WVF corresponding to injection history (Fig. 4C–F). At the same time, expansion of the pressurized Ellenburger unit results in compression (negative $\Delta \sigma _{kk}/3$) adjacent basement rocks near Inj5 for all stages of Inj5 operation.

### Role of WVF permeability

As our findings from generic studies suggest, fault permeability can be one of the main geological parameters to control the dominant mechanism inducing earthquakes along the fault. We perform a parametric study with variation in the WVF permeability, by implementing one order of magnitude larger or smaller permeability values than one from the reference case, to analyze how the fault permeability will influence pressure and stress fields and the spatio-temporal patterns of seismic events along the fault. Note that the range of permeability variation is selected based on the estimated permeabilities of damaged fault zone from laboratory testing due to lack of site-specific measured data^41^. Temporal evolution of $\text{ log}_{10}R_\sigma$ along the fault line indicates that pore-pressure diffusion will be the dominant mechanism initiating slip of the fault zone, hydraulically connected to the Ellenburger unit (Fig. 5). Less permeable WVF ($\kappa _f\times 0.1$) will restrict pore-pressure diffusion within the fault zone bounded by high-permeability injection unit and shallow basement, but poroelastic deformation can perturb stress states on the deep fault zone (Fig. 5B). Note that poroelastic stressing enhances the stability of WVF at given faulting stress regime. On the other hand, more permeable WVF ($\kappa _f\times 10$) accelerates intense perturbations in pore pressure fields along the entire fault zone, and thus, the fault stability will be controlled by the diffusion process according to the injection operations (Fig. 5C).

The static changes in Coulomb stress indicates that lower permeability limits combined effects of diffusion and poroelastic stressing, which constrains the largest $\Delta \tau$ at the fault bounded by the uppermost basement rocks (Fig. 6A). Slow, but gradual, diffusion of pore pressure over time generates along the fault at relatively shallow depths (Fig. 6B), and thus, increases in *R* are observed above the depth of $\sim 4.5$ km (Fig. 6C). More permeable WVF will allow faster and longer-range diffusion of pore pressure into the fault, consequently developing larger positive $\Delta \tau$. A series of injection through multiple wells between 2011 and 2015 can cause intense accumulation of pore pressure and elastic energy within a finite fault zone bounded by low-permeability basement rocks, which will maintain positive $\Delta \tau$ along the entire fault consistently (Fig. 6D). However, subsequent periodic reduction of injection volumes since 2015 lessens $\Delta \tau$ and generates negative $\dot{\Delta \tau }$ along the diffusion-dominant fault zone over time (Fig. 6D,E), which leads to almost no changes in the seismicity rate (Fig. 6F).

Our result shows that the fault permeability plays a significant role in determining the regime of dominant mechanisms inducing seismicity along the fault by controlling the diffusive growth of the pressurized zone and corresponding stress transfer as poroelastic response. Steady pore-pressure diffusion and poroelastic stressing can generate positive stressing rate consistently over time, which will induce more frequent seismic events along the deep fault zone. If diffusion dominates slip of the entire fault zone, rapid accumulation or reduction of pore pressure along the fault can nucleate more earthquakes, including medium-to-large magnitude events, or eliminate the potential of induced earthquakes promptly, corresponding to the operation history. The geometric capacity of fault is another governing parameter to determine the extent of pressurized region and corresponding stressing rate within a fault (e.g. smaller faults, bounded by low-permeability rocks, require less diffusion to generate the same level of $\Delta \tau$). In addition to overall hydrogeological features of the seismogenic fault zone, internal geometric complexity (e.g., presence of hydraulic pathways formed by chemical reaction or stratigraphic juxtaposition^42^) within a fault or permeability evolution as a function of effective stress acting on the fault^43^ may cause migrating of seismic events with local concentration of seismic swarms along the fault.

### Estimate of maximum earthquake magnitude

The total volume of fluid injected into the target formation ($\Delta V$) may constrain the upper limit of magnitude for induced earthquakes^44^, which can be related to the seismic moment considering poroelastic coupling effects^26^ as follows:

2 $$\begin{aligned} M_0 = \frac{(1.5-b)}{b}\frac{2f}{S_\epsilon }\Delta V, \end{aligned}$$

where the inverse of the constrained specific storage (refer to the equation 5) is the Biot’s modulus, *b* is from the G-R frequency-magnitude relation (assuming $b=1.38$ in this study, a mean value from Table 1 in^45^), and *f* is the fault frictional coefficient.

By correlating the fault geometry to mechanical deformation, the seismic magnitude can be correlated to the total amount of displacement on the fault during the seismic event. The seismic moment on a fault plane can be measured by surface integration of total displacement over the fault plane area:

3 $$\begin{aligned} M_0 = G_f\int \mathbf {u}dA, \end{aligned}$$

where, $\mathbf{u}$ [m] is the total displacement along the fault surface and *A* [m$^2$] is the rupture area which is assumed to be identical to the size of WVF surface because the whole WVF has been stressed by direct diffusion and/or poroelastic stressing associated with nearby well operations.

The G-R frequency-magnitude distribution of earthquakes can be expressed in terms of seismic moment $M_0$ (N m), which defines the moment-magnitude relation as follows^46^:

4 $$\begin{aligned} M_{\text{ w }} = \frac{\text{ log }M_0}{1.5}-6.06. \end{aligned}$$

Note that both approaches based on the total injected volume $\Delta V$ or static rupture area (*A*) do not account for the local characteristics of the heterogeneous fault zone (e.g., fault geometry and strength) and the variability of lithology and formation properties, neglecting the localized gradients of pore pressure and stresses driven by a series of well operations.

The volume-based estimate (2) gives the maximum magnitude of 5.33 (equivalent to max($M_0$) = 1.23$\times$10$^{17}$ N m) with total injected volume of 3.7$\times$10$^7$ m$^3$ from 2007 to 2020 at the Venus site. On the other hand, using the equation (3) based on the rupture area and average total displacement along the WVF, the maximum magnitude is estimated to be 3.82 (equivalent to max($M_0$) = 6.65$\times$10$^{14}$ N m) that is close to earthquake magnitudes larger than 3.0 observed near Venus, TX. This result suggests that the volume-based prediction may overestimate the earthquake magnitude, such that the site-specific fault characteristics (e.g., hydrogeological and mechanical properties, fault geometry favorable to the fault instability, and/or the presence of fractures/faults directly connecting to the injection interval) need to be considered for the risk assessment of seismic hazards.

## Conclusion

The presence of preexisting basement fault(s) intersecting a injection formation may indicate the higher potential of earthquake nucleation along the fault, but spatio-temporal migrating patterns of induced seismicity will be determined by geological and operational parameters. Our generic studies and field-scale simulation for 2015–2018 Venus earthquake sequences reveal that

- Fault permeability is one of the critical hydrogeological parameters to determine the rate of pressure diffusion into the fault and corresponding poroelastic response within/outside the pressurized region, consequently controlling spatio-temporal evolution of dominant mechanisms (pore-pressure diffusion to poroelastic stressing, or vice versa).
- Slow diffusion along a low-permeability fault or rapid release of pressure buildup within a high-permeability fault zone, caused by curtailing injection volume/duration or extraction, will limit perturbations in Coulomb stressing rate, consequently inhibiting migration of earthquake nucleation into deeper depths.
- Defining the rupture area based on site-specific fault characteristics is essential to enhance the accuracy of earthquake-magnitude prediction.

Field-scale understanding of the spatio-temporal evolution of induced earthquake sequences will provide an insight to develop optimal operations with the aim of mitigating induced seismicity for future subsurface energy activities. Furthermore, it is necessary to identify/characterize preexisting and reactivated faults precisely corresponding to perturbations in their hydraulic or mechanical characteristics by sufficient monitoring of micro-seismic events during/after well operations^47^ as well as geological or geophysical surveys, aided by statistical methodology^48^ or machine learning^49^, even prior to the operations.

## Material and methods

### Governing equations

The governing equations of linear poroelasticity can be derived with the following assumptions: (1) the medium is porous, linear elastic; (2) fluid mass is conserved; and (3) saturated fluid flow follows Darcy’s law. The flow variable (pore pressure *p*) and mechanical response (displacement field $\mathbf{u}$) are calculated simultaneously through a system of equations as follows^50–53^:

5 $$\begin{aligned}&S_{\epsilon ,i}\dot{p}-\nabla \cdot \Lambda _i\nabla p+\alpha _i\nabla \cdot \dot{\mathbf {u}}=0, \end{aligned}$$

6 $$\begin{aligned}&\nabla \left( \lambda _i+G_i\right) \nabla \cdot \mathbf {u}+\nabla \cdot G_i\nabla \mathbf {u}-\alpha _i\nabla p = \mathbf {r}, \end{aligned}$$

where $S_\epsilon$ [Pa$^{-1}$] is the specific storage at constant strain, representing the fluid volume change per unit control volume per pressure change while holding the control volume constant, $\Lambda \equiv \kappa /\eta$, defined by the permeability $\kappa$ [m$^2$] and the fluid viscosity $\eta$ [Pa s], is the flow mobility respectively. $\lambda (\equiv 2G\nu /(1-2\nu )$ [Pa], where $\nu$ [-] is drained Poisson’s ratio) and *G* [Pa] are the Lam$\acute{e}$ elastic parameters, and $\alpha$ [-] is the Biot-Willis coefficient representing the ratio of changes in the fluid volume to the total bulk volume for deformation at constant pore pressure. The source term $\mathbf{r}$ is a body force per unit bulk volume. The subscript *i* indicates each layer and fault. Two-way poroelastic coupling is defined by the presence of $\nabla \cdot \mathbf{u}$ in the flow equation (5) and $\nabla p$ in the force balance equation (6), acting as body forces in the stress equilibrium. The transient flow equation (5) can be expressed in terms of increment of fluid content $\zeta$:

7 $$\begin{aligned} S_i\dot{\zeta }-\nabla \Lambda _i\nabla \zeta -\Lambda _i\frac{\alpha (1-2\nu )}{2G(1-\nu )}F_{k,k}=0, \end{aligned}$$

where $F_{k,k}\equiv \partial F_k/\partial x_k$ is the derivative of body force per unit bulk volume, and increment of fluid content can be related to means stress ($\sigma _{kk}/3$) and pore pressure as follows:

8 $$\begin{aligned} \zeta = \frac{3\alpha }{3\lambda +2G}\left( \frac{\sigma _{kk}}{3}+\frac{p}{B}\right) , \end{aligned}$$

where *B* is the Skempton’s coefficient defined as the ratio of pore-pressure change to applied stress change for undrained condition. Solving the flow equation (5) independently of the stress field reduces to the uncoupled system, widely used in hydrological model, as follows:

9 $$\begin{aligned} S_i\dot{p}-\nabla \cdot \Lambda _i\nabla p=0, \end{aligned}$$

where $S_i$ [Pa$^{-1}$] is the uniaxial specific storativity defined under the conditions of uniaxial strain ($\epsilon _{11}=\epsilon _{22}=0$) and constant vertical stress ($\sigma _{33}=c$). In a homogeneous domain, the diffusivity for $\zeta$ in (7) and hydraulic diffusivity in (9) can be expressed in terms of poroelastic coefficients as follows^52^:

10 $$\begin{aligned} D=\Lambda \frac{\left( \lambda _{u}-\lambda \right) (\lambda +2G)}{\alpha ^2\left( \lambda _{u}+2G\right) }=\frac{\Lambda }{S}, \end{aligned}$$

### Coulomb stress change

Assuming that the fault is critically stressed, the change in total Coulomb stress ($\Delta \tau (\mathbf{x} ,t)$) from the initial state of $\Delta \tau (\mathbf{x} ,0)=0$ is correlated to the potential of induced earthquakes on the fault at a given stress state and operation scenarios^6,23,31,54^. Using positive convention for extension and pore-pressure increase, rearranging the Coulomb stress change $\Delta \tau$ leads to the following expression:

11 $$\begin{aligned} \Delta \tau (\mathbf {x},t)=[\Delta \tau _s(\mathbf {x},t)+f\Delta \sigma _n(\mathbf {x},t)]+f\Delta p(\mathbf {x},t). \end{aligned}$$

The effect of poroelastic stressing and pore-pressure diffusion on $\Delta \tau$ is evaluated using two terms: the sum of the shear and normal stress components ($\Delta \tau _s+f\Delta \sigma _n$, where *f* is the fault friction coefficient) and pore pressure change ($f\Delta p$), respectively. Positive values of $\mathrm {\Delta }\tau$, $\mathrm {\Delta }\tau _s$, and $\mathrm {\Delta }\sigma _n$ imply that the fault plane is moved closer to failure, the change in shear stress favors failure in the expected slip direction of the fault, and an increase in relative tension across the fault, respectively.

### Seismicity rate prediction

To relate the changes in Coulomb stress to the number of induced earthquakes of a given magnitude, the empirical approach developed by^55^ is expanded in terms of the Coulomb stressing rate ($\dot{\tau }$)^12,23^:

12 $$\begin{aligned} \frac{dR}{dt}=\frac{R}{t_a}\left( \frac{\dot{\tau }}{\dot{\tau _0}}-R\right) , \end{aligned}$$

where *R* [-] is the seismicity rate relative to an assumed prior steady-state seismicity rate at a background stressing rate $\dot{\tau }_0$ [MPa/yr] and given characteristic relaxation time for seismicity to restore to steady state $t_a\equiv a\bar{\sigma }/\dot{\tau }_0$ [yr], where *a* [-] is the fault constitutive friction parameter quantifies the “direct effect” in the rate-state friction law and $\bar{\sigma }$ [MPa] is the background effective normal stress acts on the fault plane. Note that the equation (12) has no threshold stress limiting the type of seismicity rate change, such that it can solve either steady state or Omori-type decay proportional to inverse of time^56^ following a rapid stress change, or both simultaneously corresponding to $\dot{\tau }(\mathbf{x} ,t)$. The seismicity rate distribution along a given fault over time $R(\mathbf{x} ,t)$ will quantify the poroelastic coupling effects on the patterns of induced seismicity for different scenarios of fault characteristics. The details of the numerical procedure can be found in^23^.

### Geologic model setting for 2015–2018 Venus earthquakes

Our field-scale model simulates hydro-mechanical behaviors of a seismogenic fault and bounding formations driven by injection operations through five SWD wells in northeast Johnson County near Venus, TX. The stratigraphic boundaries and fault geometry are identified by seismic reflection data from^29^, and the earthquake locations and magnitudes are from the Venus catalog detected in a local seismic network deployed by Southern Methodist University (SMU) and the University of Texas Institute for Geophysics (UTIG)^57^.

Figure 2A shows a map view of fault locations and 2015–2018 seismic events, colored by depth of earthquake occurrence. The water-saturated Ellenburger formation has been selected for the permanent disposal of the produced saltwater from the Barnett Shale due to its large storage capacity and high permeability of this formation^58,59^. In the Venus area, the top of the Ellenburger formation is located approximately at 2.75 km of depth, and the unit thickness is $\sim 1.15$ km, derived from the time-migrated, depth-converted seismic reflection data^29^. In addition, the seismic reflection data image two major faults, Western Venus Fault (WVF) and Eastern Venus Fault (EVF), but this modeling study focuses on the WVF, where the active seismic sequence has been observed since 2015. This seismic sequence extends from the injection unit (Ellenburger) to the basement ($\sim$2.6 to 6.1 km of depth; Fig. 2B) with orientation of N205$^\circ$/50$^\circ$NW favorable to normal faulting.

The earthquake catalogs on the cross-section along line A–A”, colored by sequential order (red for newer events), show that a majority of small-to-moderate magnitude earthquakes ($M_{\text{ w }}\le$2) are observed within the basement ($\sim$4 to 6 km of depth), not within the injection unit, and newer events occurred at deeper depth (Fig. 2B). The consecutive swarms of seismic events may represent the growth of activated fault zone depending on gradual pressurization and/or elastic stress transfer caused by injection^28^.

Since the onset of SWD operations in northeast Johnson County in 2006, five SWD wells (Inj1 to Inj5) have been operated within 100 km$^2$ of earthquakes sequences in Venus (well locations are indicated in Fig. 2A). As of October 2020, $3.68\times 10^7$ m$^3$ ($\approx 231.7$ MMbbl) of saltwater was injected through the wells, completed within the Ellenburger layer, with different start times and histories since 2007 (Fig. 2D; injection data are available at the Texas Railroad Commission (TRC) website).

Analysis of injection data from these wells as well as proximity to the WVF indicate that injection through Inj2 (red; shut-in as of 2020) and Inj4 (magenta; active injection as of 2020) may accumulate fluid volumes in the Ellenburger layer, which supports the hypothesis that continuous SWD increased pore pressure for nearly a decade, promoting failure on the deep fault zone associated with the Venus earthquake sequences. In addition, injection through Inj5 (green; active injection as of 2020) far away from the WVF may cause poroelastic stressing that can transfer elastic energy to the fault, potentially influencing spatio-temporal patterns of induced seismicity, which is supported by temporal match between earthquakes and injection history in Fig. 2C,D).

The numerical domain consists of five layers (overburden, Marble Falls limestone, Barnett shale, Ellenburger dolomite, and basement) and one main fault (WVF) in a 3-D cubic domain with dimension of 15 km [L]$\times$15 km [W]$\times$10 km [H] (Fig. 2E). The fault area is defined as $\sim 23$ km$^2$ (5 km [L]$\times$4.6 km [H]) based on the seismic reflection data and hypocenter locations^29^, and the fault zone with a width of 10 m is assumed to be hydraulically conductive and connected to the injection unit. The top boundary of the domain has no-flow and fixed conditions, whereas the remaining boundaries have constant pressure and roller boundary conditions. The material properties of each layer and frictional properties of the WVF are given in Table 2. The reference model implements hydrological and mechanical properties from previous modeling studies of earthquakes in Azle, TX^5,60^, assuming that layered sequences extends laterally to the Venus area as a part of Bend Arch-Fort Worth Basin^29^. The WVF permeability is selected as a geological parameter related to migrating pattern of seismicity, but remaining uncertainty of other material properties requires further sensitivity tests. The positive *y*-axis is considered as north, and the direction of maximum horizontal stress is N20$^\circ$E.

**Table 2:** Parameters used in the field-scale model for Venus earthquakes.

Poroelastic and transport properties$^{*}$							
	Overburden	Marble Falls	Barnett	Ellenburger	Basement	Fault	Fluid$^{\dagger }$
$\kappa$ [m$^2$]	1.88$\times$10$^{-13}$	1$\times$10$^{-17}$	1$\times$10$^{-20}$	2.96$\times$10$^{-14}$	1$\times$10$^{-19}$	2.49$\times$10$^{-15}$	–
$\phi$ [-]	0.2	0.2	0.06	0.055	0.05	0.1	–
*G* [GPa]	6	25	16.3	25	16.9	16	–
$\lambda$ [GPa]	4	16.7	13.8	16.7	19.9	16	–
$\lambda _u$ [GPa]	6	25	16.3	25	25.4	24	–
$\alpha$ [-]	0.79	0.7	0.35	0.61	0.27	0.5	–
$\rho$ [kg/m$^3$]	2600	2160	2350	2840	2750	2500	1031
$\eta$ [Pa s]	–	–	–	–	–	–	1.1$\times$10$^{-3}$
$c_f$ [1/Pa]	–	–	–	–	–	–	4.6$\times$10$^{-10}$
Thickness [km]	2.2	0.35	0.2	1.15	6.1	–	–
Friction properties and stress state of the fault$^{*}$							
*f* [-]		0.6					
*a* [-]		0.003					
$\bar{\sigma }$ [MPa]		13.3					
$\dot{\tau }_0$ [MPa/yr]		5$\times$10$^{-5}$					
$t_a$ [yr]		800					
Direction of $S_{H,max}$		N20$^\circ$E					
SWD well information$^{\ddagger }$							
		Inj1	Inj2	Inj3	Inj4	Inj5	
API Well No.		42-251-30843	42-251-32402	42-251-32450	42-251-31305	42-251-34121	
Top depth [m]		2607.56	3462.53	2847.14	3369.87	2743.20	
Bottom depth [m]		3461.31	3875.84	3619.80	3870.10	3962.40	

$*$Poroelastic and transport properties of formation and fault from previous studies of Azle earthquakes^5,60^.

$\dagger$Fluid properites are from^64^.

$$\ddagger$$Refer to^29^.

The finite-element analysis is performed using COMSOL Multiphysics 5.4^61^, and cubic and tetrahedral elements are assigned for spatial discretization of the fault and extra formations, respectively (subplot of Fig. 2E). Mesh was highly refined near the fault boundaries and injection lines to resolve the strong pressure gradients driven by the contrast of material properties and variation in injection rates. The numerical simulation runs 168 months from January 2007, when SWD operation started through Inj1, to January 2021, even though this study focuses on 2015–2018 earthquakes, which enables to obtain initial pressure and stress states in 2015 formed by preceding SWD operations. Injection operations through five SWD wells are simulated by line sources, based on depths of well completion given in Table 2.

The seismicity rate estimate implements constitutive parameter $a=0.003$, as measured in friction experiments^62^, and effective normal stress $\bar{\sigma }=13.3$ MPa at a depth of about 1 km for a rock density of 2500 kg/m$^3$. The background stressing rate $\dot{\tau }_0$ is set to $5\times 10^{-5}$ MPa/yr, based on parameter values used in previous seismicity-rate models for Texas earthquake sequences^39,63^. This parameter setting leads to a characteristic time $t_a=800$ years.

## Supplementary Information

Supplementary Information.
