# Author Correction: Permeability-controlled migration of induced seismicity to deeper depths near Venus in North Texas

**DOI:** 10.1038/s41598-022-10444-0

**Published:** 2022-04-19

**Authors:** Kyung Won Chang, Hongkyu Yoon

**Affiliations:** 1 Geotechnology and Engineering Department, Sandia National Laboratories, Albuquerque, 87123 USA; 2 Geomechanics Department, Sandia National Laboratories, Albuquerque, 87123 USA

Correction to: *Scientific Reports* 10.1038/s41598-022-05242-7, published online 26 January 2022

The original version of this Article contained an error in the copyright line.

“© The Author(s) 2022”

now reads:

“© National Technology & Engineering Solutions of Sandia, LLC 2022”

The original Article has been corrected.

