# Supplementary material for: Permeability-controlled migration of induced seismicity to deeper depths near Venus in North Texas
Source: Sci Rep. 2022 Jan 26;12:1382. doi: 10.1038/s41598-022-05242-7 (PMC8792014; doi:10.1038/s41598-022-05242-7)
Supplement: Supplementary file 1 — Supplementary Information. [file 41598_2022_5242_MOESM1_ESM.pdf]

Supplementary Materials

Title: Permeability-controlled migration of induced seismicity to deeper depths near Venus in North Texas.

Kyung Won Chang[1], Hongkyu Yoon[2].

1 Sandia National Laboratories, Geotechnology & Engineering Department, Albuquerque, 87123, USA.

2 Sandia National Laboratories, Geomechanics Department, Albuquerque, 87123, USA.

S1. Hydro-mechanical coupling effects

Figure S1A shows a 2-D homogeneous domain that represents the aerial-view of horizontal section crossing the injection point. Fluids are injected at a constant rate for 30 days through a point at the center of the model domain. The domain boundaries impose constant pressure and roller conditions, but the extensive length (5 km from the center injection point $\gg$ 1.7 km of characteristic diffusive length $\sqrt{4D_a\Delta t}$, where D_a is formation diffusivity) enables to minimize the boundary effect on the hydrological and mechanical behaviors of the formation. To obtain changes in shear and normal tractions driven by injection operation, this model assumes that faults pose the same properties to the background medium, and also, are uniformly distributed throughout the domain with orientation of N-S striking and 60°NE.

Figures S1B and S1C show $\Delta\tau$ computed along the horizontal and vertical line in a homogeneous/isotropic domain, indicated by red and blue lines in a horizontal model scheme of Figure S1A. Black horizontal dash lines represent the end of injection ($\Delta t = 30$ days). The yellow contour line of $\Delta\tau = 0.3$ MPa along the x direction indicates abrupt increase of $\Delta\tau$ at ~ 0.5 km away from the injection point after shut-in, which results from combined effects of delayed pore-pressure diffusion and instantaneous releases of poroelastic stress (Figure S1B). Neglecting poroelastic coupling effects (so-called as “uncoupled system"), pore-pressure diffusion is the only mechanism to perturb the stress state, such that $\Delta\tau \approx f\Delta p$, which results in further propagation of pore pressure and slower retardation of pressure fronts after shut-in, but no post shut-in spike in $\Delta\tau$ (white contour in Figure S1B). Along the y direction, larger slip-favorable shear traction (larger positive $\Delta\tau_s$) and less compression (smaller negative $\Delta\sigma_n$), acting on the ideally-oriented faults, generate larger $\Delta\tau$ during injection, but no spike in $\Delta\tau$ is observed after terminating injection due to immediate reduction in shear traction and slower release of compression.

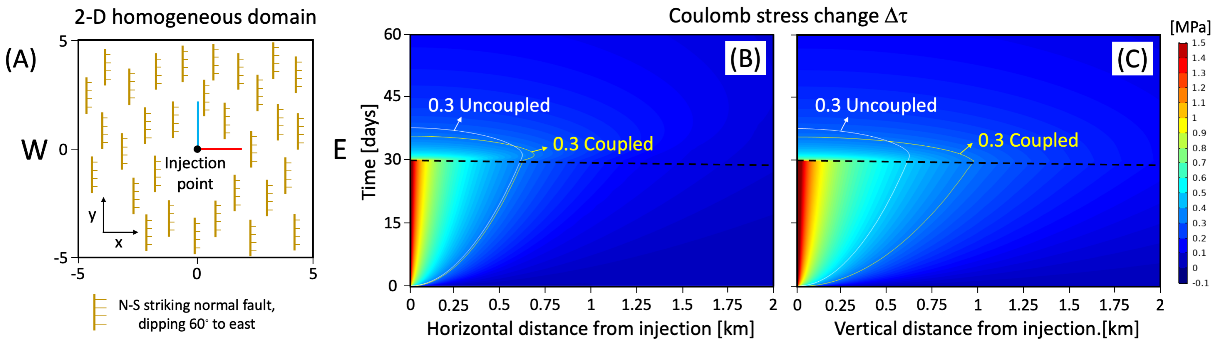

Figure S1. (A) Schematic description of the 2-D generic model domain. To compute shear and normal tractions, faults (N-S striking and dipping 60° to east) are assumed to be uniformly distributed throughout the domain; hydrological and mechanical properties of the faults are the same as the background medium. (B and C) Coulomb stress change along the horizontal and vertical lines in a 2-D aerial-view domain (red and blue line shown in Figure S1A, respectively) over time for coupled and uncoupled models (yellow and white lines, respectively).

S2. Impacts of formation rigidity on poroelastic mechanism

Figure S2 shows that spatio-temporal distribution of changes in Coulomb stress components and $\log_{10}R_\sigma$ from the reference case ($G = 7.6$ GPa) and more rigid formation one ($G = 76$ GPa), respectively. Note that both cases implement the same permeability ($\kappa = 1 \times 10^{-14}$ m^2). A more rigid formation requires more elastic strain energy for mechanical

behaviors that generates stronger and quicker poroelastic response to injection and subsequent shut-in: larger positive $f\Delta p$ and negative $\Delta\tau_s + f\Delta\sigma_n$ during injection, and rapid reduction of both quantities immediately after shut-in (Figures S2E and S2F). As a result, larger rigidity causes larger $\Delta\tau$ during injection and faster relief of $\Delta\tau$ after shut-in (Figure S2G). Due to high-permeability of the formation, faults can be destabilized dominantly by diffusion mechanism for both cases, but larger shear modulus causes faster poroelastic response to operational phases (less $\log_{10}R_\sigma$; Figure S2H). In a hydrogeological sense, larger rigidity is consistent to less storage capacity of the formation, such that injecting the same amount of fluids will cause larger pressure increases.

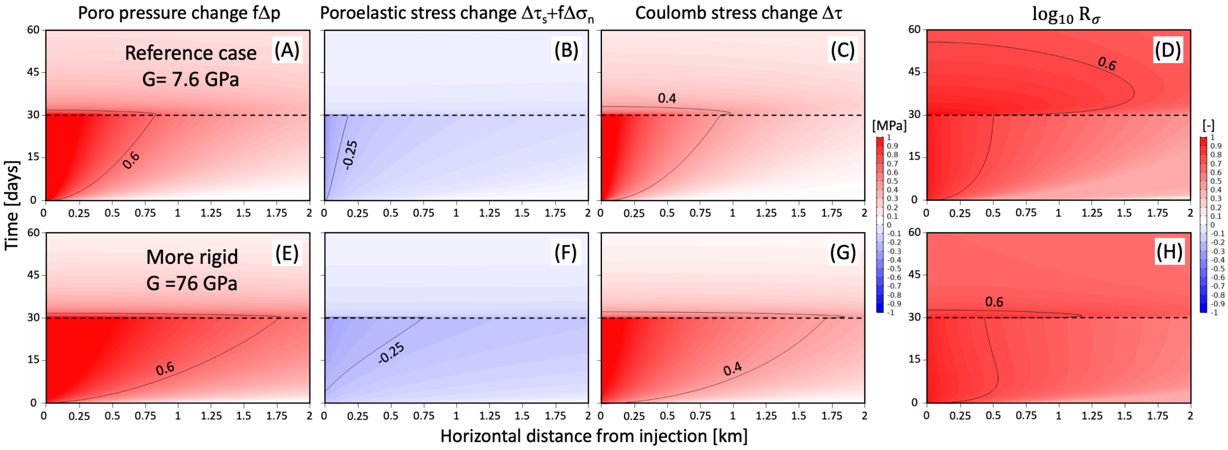

Figure S2. Spatio-temporal distribution of $\log_{10}R_\sigma$ with variation in shear modulus: (A ot D) $G = 7.6$ GPa and (E to H) $G = 76$ GPa of more rigid formation. The contour of $\log_{10}R_\sigma = 0$ represents $f\Delta p = \Delta\tau_s + f\Delta\sigma_n$, such that diffusion and poroelastic stressing contribute equally to total changes in Coulomb stress.

Supplementary materials

Supplementary Text

Figures S1 to S2
